# Early IL-1 inhibition and long-term remission in childhood-onset Still's disease: a real-world cohort study

**DOI:** 10.3389/fped.2026.1862030

**Published:** 2026-06-15

**Authors:** Burcu Bozkaya Yücel, Seyda Dogantan, Semanur Ozdel, Özlem Aydoğ

**Affiliations:** 1 Department of Pediatric Rheumatology, Samsun City Hospital, Samsun, Türkiye; 2 Department of Pediatric Rheumatology, Basaksehir CAM and Sakura City Hospital, Istanbul, Türkiye; 3 Faculty of Medicine, Department of Pediatric Rheumatology, Samsun University, Samsun, Türkiye; 4 Department of Pediatric Rheumatology, Maslak ACIBADEM Hospital, Istanbul, Türkiye

**Keywords:** anakinra, childhood-onset Still's disease, IL-1 inhibition, macrophage activation syndrome, real-world study, treatment-free remission

## Abstract

**Background:**

Childhood-onset Still's disease is a severe autoinflammatory disease characterized by systemic inflammation and risk of macrophage activation syndrome (MAS). Early cytokine-targeted therapy, particularly interleukin-1 (IL-1) inhibition, has been proposed to modify disease trajectory and improve long-term outcomes.

**Methods:**

We conducted a single-center retrospective cohort study of children with childhood-onset Still's disease followed between January 2018 and December 2025. Clinical inactive disease (CID) was defined according to Wallace criteria. Treatment patterns, biologic switching, MAS occurrence, biomarker normalization, and treatment-free remission were evaluated.

**Results:**

Eighteen patients were included (50% female), with a median age at symptom onset of 6.5 years. First-line biologic therapy consisted predominantly of IL-1 inhibition [94.4% (*n* = 17)]. Biologic switching occurred in 77.8% (*n* = 14) of patients, primarily due to treatment burden related to daily injections. CID was achieved in 11.1% (*n* = 2) of patients at 1 month, 66.7% (*n* = 12) at 3 months, and 94.4% (*n* = 17) at 6 months. MAS developed early in 44.4% (*n* = 8) of patients, with no recurrent MAS episodes during follow-up. Among MAS patients, concomitant therapies included intravenous pulse methylprednisolone in all, IVIG in 62.5% (*n* = 5), plasmapheresis in 37.5% (*n* = 3), and cyclosporine in 62.5% (*n* = 5). At last follow-up, 66.7% (*n* = 12) of patients achieved treatment-free remission. The median duration of biologic therapy prior to discontinuation was 14.5 months (IQR: 12.0–18.0). A transient discrepancy between physician and patient/parent global assessments (“perception lag”) was observed during early treatment.

**Conclusion:**

Early IL-1-targeted therapy was associated with rapid disease control, effective corticosteroid sparing, and high rates of treatment-free remission in childhood-onset Still's disease. These findings support early cytokine inhibition as a cornerstone of treat-to-target strategies and suggest a potential disease-modifying effect in Still's disease.

## Introduction

Childhood-onset Still's disease is a rare but severe systemic inflammatory disease, previously classified as systemic juvenile idiopathic arthritis (JIA) according to the International League of Associations for Rheumatology (ILAR) classification criteria (1), accounting for approximately 10%–20% of JIA cases yet contributing disproportionately to disease-related morbidity and mortality (2, 3). Unlike other JIA categories, Still's disease is predominantly driven by systemic inflammation rather than isolated synovitis and is characterized by quotidian fever, evanescent rash, hepatosplenomegaly, lymphadenopathy, and serositis (4–6). Laboratory findings, including leukocytosis, thrombocytosis, elevated acute-phase reactants, hyperferritinemia, and lactate dehydrogenase (LDH) levels, reflect a profound inflammatory burden. Among disease complications, macrophage activation syndrome (MAS) represents a potentially life-threatening hyperinflammatory condition and remains a major determinant of early mortality in affected children (7, 8).

Advances in immunopathogenesis have redefined Still's disease as a prototypic autoinflammatory disorder driven by dysregulated innate immunity, characterized by excessive activation of monocytes and macrophages together with overproduction of key proinflammatory cytokines, particularly interleukin-1 (IL-1) and interleukin-6 (IL-6) (9, 10). This evolving understanding has led to the conceptualization of a biphasic disease course, consisting of an early systemic phase dominated by cytokine-driven inflammation and, in a subset of patients, a later phase characterized by chronic arthritis and adaptive immune activation (11). Within this framework, the concept of a “window of opportunity” has emerged, suggesting that early targeted cytokine inhibition may alter disease trajectory and reduce the likelihood of progression to chronic refractory disease (12). Recent EULAR/PReS recommendations have further emphasized unified terminology under the concept of Still's disease and the importance of early cytokine-targeted treatment strategies (13).

Historically, treatment strategies relied heavily on high-dose corticosteroids, which, although effective in controlling systemic inflammation, are associated with substantial toxicity, including growth impairment, osteoporosis, metabolic complications, and increased infection risk (14). Conventional synthetic disease-modifying antirheumatic drugs (csDMARDs), such as methotrexate, have demonstrated limited efficacy in systemic disease, while tumor necrosis factor inhibitors have generally failed to provide meaningful benefit in this population (15). The identification of IL-1 and IL-6 as central mediators of disease pathogenesis has transformed the therapeutic landscape, with biologic agents targeting these pathways demonstrating rapid disease control, steroid-sparing effects, and improved clinical outcomes in both clinical trials and real-world studies (16–18). In selected centers and patient populations, IL-1 blockade may also be used as monotherapy as part of evolving treat-to-target strategies.

Despite these advances, several important challenges remain. MAS continues to occur in a substantial proportion of patients, often early in the disease course (8, 19). Previous cohorts have reported MAS frequencies ranging from 10% to 40%, whereas the relatively high frequency observed in our cohort may reflect referral bias to a tertiary care center and heightened awareness and earlier recognition of systemic inflammatory complications (7, 8, 19). In addition, real-world experience suggests that biologic switching is frequent and may be driven not only by insufficient response or adverse events but also by treatment burden, particularly in the setting of daily subcutaneous therapies in young children (20). Real-world observational studies are therefore critical to complement clinical trial data (21), particularly for evaluating early biologic intervention strategies within routine clinical practice. In this context, the assessment of clinical inactive disease (CID), durability of remission, achievement of treatment-free remission, and prevention of chronic refractory disease represents clinically meaningful outcome measures reflecting both short-term disease control and long-term disease modification (22).

In this study, we aimed to evaluate the timing of CID, rates of treatment-free remission, biologic switching patterns, and MAS occurrence in children with childhood-onset Still's disease managed with early biologic therapy in a real-world clinical setting.

## Methods

### Data collection and variable definitions

Clinical and laboratory data were retrospectively extracted from electronic medical records using a standardized data collection form. To ensure data integrity and confidentiality, all patient identifiers were removed and each subject was assigned a unique study identification number prior to analysis.

Demographic and time-dependent variables included date of birth, date of symptom onset, date of childhood-onset Still's disease diagnosis, and date of last follow-up visit. From these dates, age at symptom onset, age at diagnosis, diagnostic delay (defined as the interval between symptom onset and diagnosis), and follow-up duration (defined as the interval between diagnosis and last visit) were calculated.

### Clinical and laboratory assessments

Baseline clinical manifestations at diagnosis included fever and its duration, evanescent rash, arthritis, lymphadenopathy, hepatosplenomegaly, and serositis. Disease activity at baseline and during follow-up was assessed using Physician Global Assessment and Patient/Parent Global Assessment measured on a 0–10 cm visual analogue scale (VAS).

Physician Global VAS and Patient/Parent Global VAS were analyzed independently to capture the relationship between physician-assessed disease activity and patient-perceived well-being, particularly during the systemic-to-articular transition of Still's disease.

CID was defined according to the provisional criteria proposed by Wallace et al. (23).

Baseline laboratory parameters included complete blood count, erythrocyte sedimentation rate (ESR), C-reactive protein (CRP), serum ferritin, lactate dehydrogenase (LDH), fibrinogen and triglycerides, when available. Imaging data, including echocardiography and chest imaging, were reviewed when performed based on clinical indications, particularly to assess serositis or cardiopulmonary involvement.

### Treatment characteristics

Treatment data were recorded in detail and included the use of intravenous pulse methylprednisolone, oral corticosteroids (including duration of maintenance and tapering), and biologic therapies (anakinra, canakinumab, or tocilizumab).

Information regarding initial biologic choice, duration of first-line biologic therapy, biologic switching (timing, reason for switch, and subsequent agent), and concomitant use of conventional synthetic disease-modifying antirheumatic drugs (csDMARDs) was collected.

Adjunctive therapies, including intravenous immunoglobulin (IVIG) and plasmapheresis, were also recorded, particularly in patients with severe systemic inflammation or MAS.

The study specifically focused on real-world treatment dynamics, including biologic switching, treatment burden, and patient-related factors influencing therapeutic decisions.

### Outcome measures

The primary outcome was achievement of CID, defined according to the provisional criteria proposed by Wallace et al. (23), including absence of active arthritis, absence of systemic features attributable to Still's disease, absence of active uveitis, physician global assessment of disease activity of zero, and no evidence of active systemic inflammation (including normalization of ESR and/or CRP).

Systemic disease activity was further evaluated using inflammatory biomarkers, including CRP, ESR, and ferritin, with particular focus on time to normalization as an indicator of treatment response. CID status was assessed at routine clinical follow-up visits and recorded as present or absent. Time to first CID was defined as the interval between diagnosis and the first visit at which all CID criteria were fulfilled.

MAS was identified using the 2016 EULAR/ACR/PRINTO classification criteria (24). Disease flare was defined as recurrence of systemic and/or articular manifestations requiring therapeutic escalation after prior achievement of CID.

Time to normalization of systemic symptoms and laboratory parameters (ESR, CRP, and ferritin) was calculated from diagnosis to the first documented normal value.

### Statistical analysis

Continuous variables were assessed for normality and summarized as median with interquartile range (IQR) or mean ± standard deviation (SD), as appropriate. Categorical variables were summarized as number and percentage.

Time-to-event outcomes, including time to first CID, were summarized descriptively based on routine clinical follow-up visits. Given the observational design and limited sample size, no formal comparative analyses between biologic agents were performed.

Missing data were reported explicitly and were not imputed. All statistical analyses were performed using IBM SPSS Statistics for Windows, Version 25.0 (IBM Corp., Armonk, NY, USA).

### Ethical considerations

The study protocol was approved by the Samsun University Non-Interventional Clinical Research Ethics Committee (GOKAEK-2026/1/4). Given the retrospective nature of the study and the use of de-identified patient data, the requirement for informed consent was waived in accordance with national regulations and international ethical standards.

We used the STROBE reporting guideline (25) to draft this manuscript, and the STROBE reporting checklist when editing.

## Results

### Baseline characteristics

A total of 18 children with childhood-onset Still's disease were included in the analysis. Baseline demographic, clinical, and laboratory characteristics are summarized in Table 1. The cohort included equal numbers of females and males, with a median age at symptom onset of 6.5 years [interquartile range (IQR) 5.1–10.3] and a median diagnostic delay of 0.66 months (IQR: 0.48–0.79). At diagnosis, all patients presented with quotidian fever, and half had arthritis, while systemic features such as rash, lymphadenopathy, and hepatosplenomegaly were common. Baseline disease activity and inflammatory markers reflected a high systemic inflammatory burden, with median erythrocyte sedimentation rate (ESR) of 86.5 mm/h, C-reactive protein (CRP) of 103.5 mg/L, ferritin of 1,961.5 ng/mL, platelet count of 404 × 10⁹/L, white blood cell count of 14.1 × 10⁹/L, hemoglobin of 10.1 g/dL, and lactate dehydrogenase (LDH) of 559 U/L (Table 1).

**Table 1:** Baseline characteristics of patients with childhood-onset Still's disease (*n* = 18).

Variable	Value
Demographic characteristics	
Female/Male, *n*	9/9
Age at symptom onset, years, median (IQR)	6.5 (5.1–10.3)
Age at diagnosis, years, median (IQR)	6.6 (5.2–10.3)
Diagnostic delay, months, median (IQR)	0.66 (0.48–0.79)
Follow-up duration, months, median (IQR)	42.9 (24.8–62.4)
Clinical features and baseline investigations at diagnosis	
Fever, *n* (%)	18 (100)
Fever duration, days, median (IQR)	17.5 (15.5–20.8)
Rash, *n* (%)	13 (72.2)
Arthritis, *n* (%)	9 (50.0)
Lymphadenopathy, *n* (%)	14 (77.8)
Hepatosplenomegaly, *n* (%)	10 (55.6)
Pericardial effusion, *n* (%)	2 (11.1)
Peritonitis, *n* (%)	1 (5.6)
Disease activity at baseline	
Patient/parent global VAS (cm), median (IQR)	5.0 (5.0–6.8)
Physician global VAS (cm), median (IQR)	5.0 (4.0–5.8)
Laboratory findings	
WBC (×10⁹/L), median (IQR)	14.1 (11.1–22.3)
Hemoglobin (g/dL), median (IQR)	10.1 (8.8–10.8)
Platelets (×10⁹/L), median (IQR)	404 (276.8–493.3)
ESR (mm/h), median (IQR)	86.5 (76.0–96.0)
CRP (mg/L), median (IQR)	103.5 (56.5–149.8)
Ferritin (ng/mL), median (IQR)	1,961.5 (1,031.3–4,213.3)
LDH (U/L), median (IQR)	559 (420.5–1,158.5)

IQR, interquartile range; VAS, visual analogue scale; WBC, white blood cell; ESR, erythrocyte sedimentation rate; CRP, C-reactive protein; LDH, lactate dehydrogenase.

### Treatment characteristics and biologic switching

Treatment exposure and switching patterns are detailed in Table 2. All patients received systemic corticosteroids at disease onset, with rapid tapering and complete discontinuation occurred within 12.5 weeks. 72.2% (*n* = 13) of patients received intravenous pulse methylprednisolone. IL-1 inhibition was used as first-line biologic therapy in nearly all patients [94.4% (*n* = 17) received anakinra]. Biologic therapy was initiated early after diagnosis, with a median time to initiation of 8.5 days (IQR: 4.3–14.0). Biologic switching was frequent [77.8% (*n* = 14)], predominantly driven by treatment burden rather than inadequate response. The majority of patients switched to canakinumab [92.9% (*n* = 13)], while only one patient switched to tocilizumab [7.1% (*n* = 1)]. Concomitant conventional synthetic disease-modifying antirheumatic drugs (csDMARDs) and adjunctive therapies such as intravenous immunoglobulin (IVIG) and plasmapheresis were used in a subset of patients, primarily in the context of severe systemic inflammation or MAS.

**Table 2:** Treatment characteristics and biologic switching patterns.

Variable	Value
Systemic corticosteroid therapy	
Any systemic corticosteroid use, *n* (%)	18 (100)
Pulse methylprednisolone, *n* (%)	13 (72.2)
Time to steroid discontinuation, weeks, median (IQR)	12.5 (10.0–19.5)
Biologic therapy	
First-line biologic: Anakinra, *n* (%)	17 (94.4)
First-line biologic: Tocilizumab, *n* (%)	1 (5.6)
Duration of first-line biologic therapy prior to switching, months, median (range)	3.0 (1–5)
Biologic switching, *n* (%)	14 (77.8)
Injection burden, *n* (%)	11 (78.6)
Insufficient response, *n* (%)	2 (14.3)
Adverse event leading to biologic switch, *n* (%)	1 (7.1)
Switch to canakinumab, *n* (%)	13 (92.9)
Switch to tocilizumab, *n* (%)	1 (7.1)
Concomitant and adjunctive therapies	
Any csDMARD use, *n* (%)	6 (33,4)
Cyclosporine, *n* (%)	5 (27.8)
Methotrexate, *n* (%)	1 (5.6)
IVIG use, *n* (%)	9 (50.0)
Plasmapheresis, *n* (%)	3 (16.7)
MAS-associated concomitant therapies (*n* = 8)	
IVIG use, *n* (%)	5 (62.5)
Plasmapheresis, *n* (%)	3 (37.5)
Cyclosporine use, *n* (%)	5 (62.5)
Biologic-related adverse events, *n* (%)	5 (27.8)

All patients who developed MAS received pulse methylprednisolone therapy. csDMARD, conventional synthetic disease-modifying antirheumatic drug; IVIG, intravenous immunoglobulin.

Biologic switching was observed in 14 patients (77.8%), most commonly due to injection burden [78.6% (*n* = 11)], followed by insufficient response [14.3% (*n* = 2)] and adverse events [7.1% (*n* = 1)]. Canakinumab was the most frequently selected alternative agent. The median duration of first-line biologic therapy prior to switching was 3.0 months (range: 1–5 months).

Biologic-related adverse events were observed in 5 patients (27.8%). One patient discontinued tocilizumab because of a cutaneous adverse reaction. Hepatotoxicity was the most frequent adverse event and occurred in four patients receiving IL-1 inhibitors, including three patients receiving anakinra and one receiving canakinumab. The patient receiving canakinumab discontinued treatment because of hepatotoxicity. Among the patients receiving anakinra, one patient discontinued anakinra with subsequent resolution of liver enzyme abnormalities. Two patients developed persistent elevation of liver function tests leading to discontinuation of anakinra. One of these patients underwent liver biopsy, which demonstrated non-specific inflammatory findings without evidence of MAS-related hepatic involvement; canakinumab and azathioprine therapy were subsequently initiated. The other patient received azathioprine therapy because of persistent immune-mediated hepatic involvement. Neither patient had concomitant MAS findings, and autoimmune hepatitis-specific autoantibodies were negative. All hepatic abnormalities resolved within three months.

Among the eight patients who developed MAS, all received pulse methylprednisolone therapy. In addition, 5 patients (62.5%) received IVIG, 3 patients (37.5%) received plasmapheresis, and 5 patients (62.5%) received cyclosporine.

Importantly, all patients who underwent biologic switching maintained CID following transition, suggesting that the switch to longer-acting biologics did not compromise clinical stability (Table 2).

### Clinical inactive disease and disease activity over time

Rates of clinical inactive disease (CID) over time are presented in Table 3. CID was achieved rapidly, with 11.1% (*n* = 2) of patients fulfilling criteria at 1 month, 66.7% (*n* = 12) at 3 months, and 94.4% (*n* = 17) at 6 months. The median time to first CID was 1.0 month (IQR: 1.0–2.0), indicating rapid disease control. Clinical improvement was reflected in a significant reduction of both Physician and Patient/Parent Global VAS scores. From baseline median values of 5.0 (IQR: 4.0–5.8) and 5.0 (IQR: 5.0–6.8), respectively, both scores declined to 0.0 (IQR: 0.0–0.0) by Month 6 (Table 3). A transient discrepancy between physician and patient/parent assessments was observed at Month 1, with Patient/Parent Global VAS scores remaining higher despite marked improvement in physician-assessed disease activity. At Month 12, complete concordance (100%) was observed between physician and patient/parent global assessments, with all patients maintaining clinical inactive disease, indicating sustained alignment between objective and patient-reported outcomes. Although disease flares occurred during longitudinal follow-up, all patients were in inactive disease at the scheduled 12-month assessment.

**Table 3:** Clinical inactive disease (CID) and disease activity at routine clinical follow-up visits.

Time Point	CID Present, *n* (%)	Physician Global VAS, median (IQR)	Patient/Parent Global VAS, median (IQR)
Baseline	0 (0)	5.0 (4.0–5.8)	5.0 (5.0–6.8)
1 month	2 (11.1)	1.8 (1.0–2.2)	2.5 (2.0–3.0)
3 months	12 (66.7)	0.5 (0.0–1.0)	0.8 (0.0–1.2)
6 months	17 (94.4)	0.0 (0.0–0.0)	0.0 (0.0–0.0)
12 months	18 (100)	0.0 (0.0–0.0)	0.0 (0.0–0.0)

Time to first CID, months, median (IQR): 1.0 (1.0–2.0). CID was assessed cross-sectionally at routine clinical follow-up visits.

CID, clinical inactive disease; VAS, visual analogue scale; IQR, interquartile range.

### Disease flares, macrophage activation syndrome, and biomarker normalization

Disease flares were observed in 38.9% (*n* = 7) of patients during follow-up, with a median time to first flare of 13.6 months (IQR: 3.0–51.0). No flares were observed in 61.1% (*n* = 11) of patients, while 27.8% (*n* = 5) experienced one flare and 11.1% (*n* = 2) two flares. MAS occurred in 44.4% (*n* = 8) of patients, typically early in the disease course (median onset 9 days, IQR: 3.8–15.0), with no recurrent MAS episodes observed during follow-up.

Resolution of systemic inflammation was reflected by the rapid normalization of acute-phase reactants: median time to CRP normalization was 18.5 days (IQR: 10.0–24.8), median time to ESR normalization was 37.5 days (IQR: 30.0–60.8), and ferritin normalization was achieved within a median of 30.0 days (IQR: 15.8–36.5) (Table 4). The rapid normalization of inflammatory biomarkers paralleled the achievement of CID, supporting effective early suppression of systemic inflammation.

**Table 4:** Disease flares, macrophage activation syndrome, and laboratory normalization.

Variable	Value
Disease flares	
Disease flares, *n* (%)	7 (38.9)
Time to first flare, months, median (IQR)	13.6 (3.0–51.0)
No flares, *n* (%)	11 (61.1)
One flare, *n* (%)	5 (27.8)
Two flares, *n* (%)	2 (11.1)
Macrophage activation syndrome	
MAS occurrence, *n* (%)	8 (44.4)
Time to MAS onset, days, median (IQR)	9 (3.8–15.0)
Laboratory normalization	
Time to CRP normalization, days, median (IQR)	18.5 (10.0–24.8)
Time to ferritin normalization, days, median (IQR)	30.0 (15.8–36.5)
Time to ESR normalization, days, median (IQR)	37.5 (30.0–60.8)

MAS, macrophage activation syndrome; CRP, C-reactive protein; ESR, erythrocyte sedimentation rate; IQR, interquartile range.

### Long-term treatment status and treatment-free remission

Long-term treatment outcomes are summarized in Table 5. At last follow-up, 66.7% (*n* = 12) of patients were in treatment-free remission. The median duration of biologic therapy prior to discontinuation was 14.5 months (IQR: 12.0–18.0). Ongoing therapy in the remaining patients included canakinumab [22.2% (*n* = 4)], tocilizumab [5.6% (*n* = 1)], or azathioprine [11.1% (*n* = 2)]. Notably, azathioprine use was limited to patients with immune-mediated hepatic involvement and was not intended for the treatment of Still's disease activity.

**Table 5:** Long-term treatment status at last follow-up.

Treatment status	*n* (%)
Treatment-free remission	12 (66.7)
Canakinumab	4 (22.2)
Tocilizumab	1 (5.6)
Azathioprine	2 (11.1)

Categories are not mutually exclusive because one patient receiving canakinumab was also treated with azathioprine for immune-mediated hepatic involvement.

## Discussion

Our study provides real-world evidence supporting the effectiveness of early cytokine inhibition, particularly IL-1 blockade, in childhood-onset Still's disease. The rapid achievement of CID in the majority of patients within the first three months, and sustained treatment-free remission in two-thirds of patients, highlight the transformative impact of biologic therapies in routine pediatric practice. These findings are consistent with previous clinical trials demonstrating the efficacy of IL-1 and IL-6 inhibitors in Still's disease (16–18), and extend these observations by demonstrating favorable rates of treatment-free remission in a real-world cohort, as also reported in early IL-1 blockade (26). Additionally, the short diagnostic delay and early initiation of biologic therapy observed in our cohort may be attributable to early referral patterns and increased awareness of systemic inflammatory diseases in a specialized tertiary care center. These findings are consistent with a potential disease-modifying effect of early IL-1 inhibition, rather than mere disease control.

Notably, our detailed analysis revealed a “perception lag” at Month 1, where the Physician Global VAS (1.8) was lower than the Patient/Parent Global VAS (2.5). In our study, we intentionally prioritized these independent scales over composite indices to better capture the systemic-to-articular transition of Still's disease. Unlike aggregate measures that can mask individual clinical nuances, decoupled VAS scores allowed us to demonstrate that biological remission—marked by the resolution of objective inflammatory markers—often precedes the family's perception of functional recovery. This perception lag may reflect factors beyond inflammatory activity, including treatment burden, injection-related distress, and functional concerns experienced by patients and families, and is consistent with previously reported discrepancies between physician- and patient-reported outcomes in JIA (27). This finding underscores that disease control in Still's disease should not be evaluated solely based on biological remission but also on treatment tolerability and its impact on patient and family quality of life.

Together, these findings highlight a close relationship between biomarker normalization and clinical remission, reinforcing the concept of early cytokine-driven inflammation and the importance of timely targeted therapy in childhood-onset Still's disease. In our cohort, the rapid normalization of CRP and ferritin within weeks closely paralleled—and in many cases preceded—the achievement of CID, indicating that effective suppression of systemic inflammation is a key driver of clinical remission in early-treated Still's disease (26, 28). While CRP normalized rapidly, ferritin demonstrated a slightly delayed but still robust decline. Notably, although ferritin normalization occurred later than CRP, the relatively rapid decline in ferritin levels in a cohort with high MAS frequency reflects a strong and effective response to early cytokine inhibition, resulting in reduced inflammation. These findings support a treat-to-target approach in early Still's disease, consistent with previous studies and recent EULAR/PReS recommendations (13, 26, 28).

The concept of a “window of opportunity” has been proposed as a critical determinant of long-term outcomes in Still's disease (12, 26). Our data support this hypothesis, showing that early initiation of IL-1 inhibition was associated with rapid disease control, effective corticosteroid sparing, and high rates of treatment-free remission. This aligns with prior observational studies suggesting that early aggressive cytokine inhibition may prevent progression to chronic arthritis and improve long-term prognosis (26, 28). Our findings provide real-world support for this concept, suggesting that it may alter disease trajectory and increase the likelihood of sustained remission. The durability of this early response is further evidenced by the 100% concordance achieved between physician and parent assessments by Month 12, where all patients maintained inactive disease. This sustained alignment further supports the potential long-term disease-modifying effect of early IL-1 inhibition (12, 26).

Notably, the 66.7% treatment-free remission rate observed in our cohort further supports the likelihood that early cytokine-targeted therapy within a treat-to-target framework can reduce long-term corticosteroid toxicity and optimize developmental outcomes (22).

MAS remains a major challenge in Still's disease, with reported frequencies ranging from 10% to 40% depending on study design and population (8, 19). In our cohort, MAS occurred in 44.4% (*n* = 8) of patients, typically early in the disease course. This relatively high frequency may reflect referral bias to a tertiary center and heightened awareness leading to early recognition rather than a true increase in incidence. Notably, despite the high frequency of MAS, affected patients were still able to achieve CID and treatment-free remission, highlighting the robustness of early cytokine-targeted therapy even in severe disease phenotypes (7).

Furthermore, treatment switching in our cohort was predominantly driven by injection burden rather than lack of efficacy, highlighting the impact of treatment-related factors on real-world therapeutic decisions. Biologic switching occurred at a high rate (77.8%, *n* = 14), further underscoring the importance of patient-centered considerations in treatment planning. This finding is consistent with previous studies demonstrating that the daily injection requirement associated with anakinra may negatively affect treatment adherence and patient satisfaction, often leading to a preference for longer-acting agents such as canakinumab (20, 29). Switching due to true treatment failure was relatively uncommon, suggesting that early cytokine-targeted therapy is generally effective in achieving disease control. Notably, all patients who underwent biologic switching maintained CID following transition to the alternative biologic agent, supporting the clinical stability of longer-acting treatment strategies. In selected centers and patient populations, IL-1 blockade may also be successfully used as monotherapy, reflecting evolving treatment approaches in Still's disease (13, 26).

Adverse events were observed in a subset of patients, with hepatotoxicity emerging as the most frequent finding. Elevated liver enzymes have been reported in association with IL-1 inhibitors such as anakinra (30, 31) and are generally considered a relatively common but typically reversible adverse effect of biologic therapies used in Still's disease. In our cohort, persistent liver enzyme elevation in two patients suggested an immune-mediated hepatic process rather than active systemic inflammation or MAS. These findings highlight that treatment success in Still's disease is influenced not only by pharmacologic efficacy but also by treatment burden, tolerability, and adherence, emphasizing the need for individualized and patient-centered treatment strategies.

The differentiation between Still's disease–related hepatopathy, subclinical MAS, and drug-induced liver injury remains challenging in clinical practice. Recent reports have described anakinra-associated hepatotoxicity ranging from transient transaminase elevation to acute liver failure in pediatric rheumatology (30, 31). In our cohort, persistent liver dysfunction despite discontinuation of anakinra, together with negative autoimmune hepatitis-specific autoantibodies and nonspecific liver biopsy findings, suggested an immune-mediated hepatic process rather than active MAS or isolated drug toxicity. The favorable response to azathioprine further supported this interpretation. Although azathioprine is not part of standard Still's disease management (13), it was used as rescue therapy in this small subset of patients with persistent hepatic involvement after exclusion of alternative etiologies, including autoimmune hepatitis (32).

Strengths of our study include the use of standardized criteria for CID (23), systematic assessment of MAS using validated classification criteria (24), and adherence to STROBE guidelines for observational studies (25). Additionally, the relatively short diagnostic delay and early initiation of biologic therapy provide insight into outcomes within a treat-to-target framework in a real-world tertiary care setting.

Limitations of this study include its retrospective design, single-center setting, and relatively small sample size. However, given the rarity of childhood-onset Still's disease, our cohort size is comparable to those reported in real-world studies. Despite these limitations, the consistency and magnitude of the treatment response across patients support the clinical relevance and robustness of our findings. The relatively high frequency of MAS may reflect referral bias and enrichment for patients with a predominantly systemic phenotype rather than true incidence. The absence of a comparator group is inherent to the real-world observational design and reflects early adoption of IL-1 inhibition as standard practice in our center. Importantly, the use of independent Global VAS assessments, rather than composite indices, provides a more comprehensive evaluation of both inflammatory activity and patient-perceived recovery within a treat-to-target framework. Notably, no patients in our cohort developed refractory disease, refractory arthritis, refractory MAS, or Still's disease–associated lung disease during follow-up, although the relatively small sample size may have limited the ability to capture these uncommon severe outcomes.

Nonetheless, our findings provide robust real-world evidence complementing randomized controlled trials and reinforcing the role of early cytokine-targeted strategies in contemporary Still's disease management. Future prospective, multicenter studies are warranted to confirm these findings and to better define optimal treatment strategies.

In conclusion, our study reinforces the role of early IL-1 inhibition as a cornerstone of childhood-onset Still's disease management, demonstrating rapid achievement of CID, effective corticosteroid sparing, and high rates of treatment-free remission. These findings support a paradigm shift toward early cytokine-targeted intervention, suggesting that timely IL-1 inhibition may not only control inflammation but also help modify long-term disease trajectory. The high rate of treatment-free remission observed in our cohort further indicates that early intervention may represent a critical threshold for long-term disease modification in Still's disease. These findings also highlight the importance of patient-centered outcomes, as the observed perception lag suggests that clinical remission may not fully reflect patient and family experience during early treatment. This study provides additional real-world evidence on early IL-1–targeted therapy and treatment-free remission in childhood-onset Still's disease, with important implications for optimizing treatment strategies in affected children.

## Data Availability

The raw data supporting the conclusions of this article will be made available by the authors, without undue reservation.
